# A Comparison of the Flow Cytometric Analysis Results of Benign and Malignant Serous Tumors of the Ovary

**DOI:** 10.3390/cancers17162691

**Published:** 2025-08-19

**Authors:** Ozgur Ozdemir, Mustafa Emre Duygulu, Yavuz Tekelioglu, Safak Ersoz, Suleyman Guven

**Affiliations:** 1 Independent Researcher, Trabzon 61050, Turkey; 2 Department of Medical Oncology, Faculty of Medicine, Karadeniz Technical University, Trabzon 61080, Turkey; meduygulu@ktu.edu.tr; 3 Department of Hystology and Embryology, Faculty of Medicine, Karadeniz Technical University, Trabzon 61080, Turkey; ytekeli@ktu.edu.tr; 4 Department of Pathology, Faculty of Medicine, Karadeniz Technical University, Trabzon 61080, Turkey; sersoz@ktu.edu.tr; 5 Department of Obstetrics and Gynecology, Faculty of Medicine, Karadeniz Technical University, Trabzon 61080, Turkey; sguven@ktu.edu.tr

**Keywords:** annexin V, flow cytometry, ovarian cancer, serous tumor

## Abstract

**Background/Objectives**: Flow cytometric analysis of cellular DNA content has prognostic importance in ovarian cancer and its measurement could contribute to clinical practice. The aim of this study was to compare serous cystadenoma and serous cystadenocarcinoma cases in terms of their flow cytometric analysis results. **Methods**: In total, 60 serous ovarian tumor cases (30 cases of ovarian serous cyst adenoma and 30 cases of ovarian serous cystadenocarcinoma) in paraffin blocks were extracted from hospital pathology archives for flow cytometric analysis. Cell cycle changes, aneuploidy ratio, and annexin V levels were compared. **Results**: The S phase cell ratio, proliferative index, aneuploidy cell ratio, and annexin V apoptotic index were significantly higher in the cancer group compared to the cystadenoma group. However, the G2/M phase cell ratio was found to be similar in both groups. When the annexin V apoptotic index cutoff value was selected as 27.65%, the sensitivity was calculated as 90.0% and the specificity as 93.3% for predicting serous ovarian carcinoma (AUC, 0.872; *p* < 0.001; 95% CI, 0.761–0.984). **Conclusions**: In serous cystadenocarcinoma cases, cell cycle activity was increased compared to serous cyst adenoma, but the similarity of G2/M activities suggested that serous cystadenocarcinoma may be a member of the “metaneoplasia” process. The detection of annexin V expression in ovarian tumors by flow cytometric analysis is a cheap, fast, and easy diagnostic method that can be used in the diagnosis of ovarian cancer. Annexin V expression is an effective biomarker with high sensitivity and specificity that could be used in the diagnosis of serous ovarian cancer.

## Simple Summary

Serous tumors of the ovary may progress from serous cystadenoma to serous cystadenocarcinoma. The detection of annexin V expression in ovarian tumors by flow cytometric analysis is a cheap, fast, and easy diagnostic method that could be used in the diagnosis of ovarian cancer. When the annexin V apoptotic index cutoff value was selected as 27.65%, the sensitivity was calculated as 90.0% and the specificity as 93.3% for predicting serous ovarian carcinoma. Annexin V expression is an effective biomarker with high sensitivity and specificity that could be used in the diagnosis of serous ovarian cancer.

## 1. Introduction

The most common cancers of the ovary are tumors that develop from the surface epithelium of the ovary. The most common of these is serous cystadenocarcinoma. It is the second most common gynecological cancer in developed countries and the third most common in developing countries. The most lethal gynecological cancer is also ovarian cancer. The average age at diagnosis is 63 years and the lifetime risk of developing ovarian cancer is 1.3%. Advanced age, endometriosis, and PCOS are some of the possible risk factors. When diagnosed, patients are usually in an advanced stage and mainly present with complaints of abdominal bloating and ascites. Diagnosis is made by pathological analysis of the surgical specimen. Treatment is surgery and chemotherapy, and the 5-year survival for stage III tumors is around 41% [1,2,3].

A dualistic model of carcinogenesis was identified for the development of epithelial ovarian cancer. Accordingly, in terms of serous tumors, type I carcinomas develop from the fallopian tube epithelium and their precursor lesions are considered atypical proliferative tumors. Type I tumors have a good prognosis and grow slowly. The best example of this group of precursor lesions is the borderline ovarian tumor. According to some authors, type I tumors start with benign lesions (cyst adenoma, cyst adenofibroma) and show morphological molecular changes over time, turning into atypical proliferation/borderline ovarian tumors and then invasive cancer [4,5]. Type II tumors generally present in an advanced stage and have a poor prognosis. Their precursor lesions are considered serous tubal intraepithelial carcinoma [6]. It is not clear where ovarian serous cystadenoma plays a role in ovarian tumor carcinogenesis.

Serous cystadenoma is the second most common benign ovarian neoplasm. It is a thin-walled, uni- or multi-loculated adnexal mass that can reach 5–20 cm in size. Diagnosis is made by pathological examination of the excised cyst. These cysts are usually asymptomatic and are detected incidentally during examination. Surgical excision is performed in cases of a ruptured cyst and acute abdomen; in cases of hemorrhage and hemodynamic instability; and in clinical situations such as an abscess, torsion, and malignancy risk. The risk of recurrence after excision is low [7].

Apoptosis is defined as programmed cell death and is a mechanism that exists for the removal of damaged and unnecessary cells. The system, which works intrinsically (mitochondrial) or extrinsically (death receptor-mediated), ultimately activates the caspase system and provides cell death in a controlled manner [8,9]. With apoptosis dysregulation, damaged cells escape from programmed cell death, proliferate, and spread uncontrollably. This situation is defined as another theory in carcinogenesis [10]. With the flow cytometric analysis of the cell cycle, it is possible to measure the change in apoptosis in the cell using DNA dyes. Apoptotic cells will take up more dye because their membrane permeability is different compared to annexin V-negative cells [11]. Annexin V is a recombinant phosphatidylserine binding protein that provides information about cell apoptosis by binding strongly and specifically to the phosphatidylserine in the membrane [12].

There is no evidence in the literature that serous cystadenoma is a precursor lesion of serous cystadenocarcinoma. However, it has been reported that low-grade tumors are precursor lesions of ovarian serous cancer. This supports the thesis that serous tumors may have precursor lesions in carcinogenesis. Specifically, the theory that lesions such as serous cystadenoma may transform into high-grade tumors with the “metaneoplasia” development process of the ovarian surface epithelium has been reported by one author [13]. In our study, flow cytometric analysis of serous adenoma and carcinoma cases was used to evaluate the possible “metaneoplasia” process.

The aim of this study was to compare serous cystadenoma cases with serous cystadenocarcinoma cases in terms of their flow cytometric analysis results.

## 2. Materials and Methods

The Karadeniz Technical University Farabi Hospital Pathology archive was scanned retrospectively. In total, 60 serous ovarian tumor cases (30 cases of ovarian serous cyst adenoma and 30 cases of ovarian serous cystadenocarcinoma) in paraffin blocks were used for flow cytometric analysis. For this retrospective study, ethics committee approval was obtained from Karadeniz Technical University Faculty of Medicine Scientific Research Ethics Committee with file number 2023/143 (date 12 October 2023).

After obtaining ethics committee permission, all cases in the pathology database were scanned one after the other retrospectively to reach 30 cases in each group, which provided sufficient statistical power for the analysis. The G*Power 3.1.9.7 computer program was used for the sample size calculation. All cases meeting the inclusion criteria were included in the study without exception.

The inclusion criteria for the serous cystadenocarcinoma group were as follows: patients who had (a) surgical staging performed according to the FIGO staging system [14] in the clinic where the study was conducted, (b) all pathological analyses performed in the relevant clinic, (c) clinical/laboratory findings that were accessible from the hospital data automation system, and (d) high-grade serous histology. Cases with other epithelial ovarian tumor pathologies, that were not fully staged, with non-epithelial ovarian cancer, with an age below 18 years, with autoimmune diseases, or with a history of immune suppressive therapy were excluded from this study.

The inclusion criteria for the serous cystadenoma group were as follows: patients who had (1) undergone surgery in the clinic where the study was conducted, (2) all pathological analyses conducted in the relevant clinic, and (3) clinical/laboratory findings that could be accessed from the hospital data automation system. Cases with other ovarian cyst pathologies (endometriosis, teratoma, etc.), with an age below 18 years, with autoimmune diseases, or with a history of immune suppressive therapy were excluded from this study.

### 2.1. Deparaffinization and Flow Cytometry

We deparaffinized ovarian tissue samples and performed the flow cytometric analysis method detailed below.

The procedure for paraffin-embedded tissue preparation enables the extraction of a single-cell suspension of cells from a block of paraffin-embedded tissue. DNA analysis can then be performed. The procedures used the following reagents: (1) RNAse solution: 3600 units/mL (a final activity of 180 units/mL for Bauer’s solutions and 0.45 gm of RNAse A (activity, 80) in 10 mL of PBS); (2) Bauer’s stain solution (90 mL of 4 mM (1.17 gm/1.0 L H_2_O) sodium buffer, pH = 7.8; 5 mL of RNAse solution; 3.0 gm of polyethylene glycol 8000 (PEG 8000); 5 mL of propidium iodide solution (50 µg/mL); 1 mL of 10% Triton X-100; adjusting the final pH to 7.2; and taking an aliquot of 1 mL/tube and freezing at −70 °C); (3) Bauer’s salt solution (4 mL of 0.4M NaCl (2.34 gm per 100 mL of H_2_O), 1 mL of 10% Triton X-100, 3.0 gm of PEG 8000, 5 mL of propidium iodide solution (50 µg/mL), stirring for five minutes, adjusting the final pH to 7.2, and taking an aliquot of 1 mL/tube and freezing at −70 °C); and (4) pepsin solution (100 H_2_O, 0.9 gm of NaCl, 0.5 gm of pepsin, mixing well after each addition, and adjusting the final pH to 1.5 with 2N HCl).

The steps for preparing ovarian tissue for analysis were completed as follows: (1) Cut three 30 gm thick sections from a paraffin block with a microtome, trim off excess paraffin, and place the sections in a 10 mL glass test tube. Use two 50 gm sections instead if the microtome cuts pieces that are this wide. (2) Add 3 mL of Histo-clear (light-sensitive; keep in a brown bottle). (3) Incubate the tissue for 10 min at RT and vortex the sample. (4) Centrifuge the solution at 600× *g* for 5–10 min. (5) Aspirate the Histo-clear supernatant. Repeat steps 2–5 (two washes in total). Vortex the sample again after each addition. (6) Add 3 mL of 100% ethanol and incubate the tissue for 10 min at room temperature. Centrifugate the solution at 600× *g* for 5–10 min and aspirate the supernatant. (7) Repeat step 6 successively with 95%, 70%, and 50% ethanol. (8) Add 3 mL of H_2_O, wash the tissue for 1–2 min, centrifugate the solution at 500× *g*, and then aspirate and discard the supernatant. Repeat this step once more (2 washes). (9) If possible, place the tissue on a slide and separate the malignant tissue from the normal tissue using a scalpel and dissecting scope. (10) Add 1 mL of 0.1% pepsin in 0.9 M NaCl (pH 1.5). Incubate the sample for 30 min at 37 °C, vortexing vigorously every 5 min. (11) Wash the cells with 1 mL of RPMI, vortex the tissue, centrifugate the sample, and decant the supernatant. Resuspend the sample in 1 mL of RPBI. (12) Filter the solution through 30 gm nylon mesh, pool similar samples, and count 5 × 10^6^ cells/mL if desired. However, the count will often be lower. Dilute CRBC to 1.5 × 10^6^/mL and add 200 µL to each tube as an internal standard. If necessary, dilute the solution with RPMI. (13) Add 1 mL of Bauer’s stain solution to each tube and vortex well. (14) Cap the tubes and incubate the sample in a 37 °C water bath for 20 min. (15) After incubation, add 1 mL of Bauer’s salt solution to each tube and mix. (16) Store at 4 °C until the tissue is ready for flow cytometry [15,16,17].

Deparaffinization was performed for paraffin-embedded ovarian tissues and then DNA dyes were used for flow cytometry and annexin V expression. First, paraffinized ovarian tissues were deparaffinized using chemical and mechanical methods [18]. Ovarian tissue samples were suspended. The suspension was then passed through a nylon DNA mesh and the particles were filtered (Spectramesh-nylon, 50-micron Backman-Coulter). The BD Pharmingen Pe ANNEXIN V Apoptosis Detection Kit was used to stain each suspension (Cat: 559763, Lot: 5306537). Flow cytometry analysis was performed with the BD Accuri C6 Cytometer device and apoptosis peak percentage rates detected in histograms were evaluated using the analysis program BD Accuri C6 Cytometer software (version 1.0.264.21).

From the DNA histogram graph obtained for all samples that were studied in the flow cytometry analyzer, the G0/G1 peak ratio, G2/M peak ratio, S phase percentage, and aneuploid cell percentage data were obtained. In addition, the proliferative index (PI) was calculated with the formula (S + G2/M)/(G0/G1 + S + G2/M) × 100.

### 2.2. Apoptotic Index

The apoptotic index was calculated by dividing the percentage of annexin V-positive cells by the total number of cells in the gate [18,19].

### 2.3. Statistical Analyses

The SPSS 23 program designed for Windows was used for statistical analysis. All continuous variables were described as the mean and standard deviation, while categorical variables were described as a percentage of the total group. All statistical tests were two-sided, and a *p*-value < 0.05 was determined as statistically significant. The conformity of the data to a normal distribution was analyzed using the Kolmogorov–Smirnov test in the SPSS environment. In the case of conformity, parametric tests were used for comparison. Student’s *t*-test, chi-square test, and Fisher’s exact test were used to compare variables. Pearson’s correlation test was used to determine the relationship between some laboratory findings. ROC analysis was used to determine sensitivity, specificity, and cut-off points.

The G*Power 3.1.9.7 computer program was used to calculate sample size. Accordingly, the effect size was accepted as 0.87. The analysis part of the mean value comparison between two independent groups was selected and, if there were 30 cases in each group, the alpha error rate was accepted as 5% and the real power as 95.40%. The input data for the power analysis were as follows: tail(s) = one, effect size d = 0.87, α err prob = 0.05, power (1-β err prob) = 0.95, and allocation ratio N2/N1 = 1. The output data were as follows: noncentrality parameter δ = 3.3694955, critical t = 1.6715528, Df = 58, sample size group 1 = 30, sample size group 2 = 30, total sample size = 60, and actual power = 0.9540002.

## 3. Results

A total of 60 serous ovarian tumor data points (30 cases of serous cystadenoma and 30 cases of serous cystadenocarcinoma) were evaluated. Since a total of 30 cases were reached in each group, the actual power of this study was 95.40% (alpha margin of error was accepted as 5%). A comparison of serous cystadenoma and carcinoma groups for select clinical data is given in Table 1. Since age–gravida parity data met the parametric test assumptions, Student’s *t*-test was used for comparison. Other demographic data did not meet the parametric statistical test assumptions. Non-parametric tests were used for comparison. Mean age was found to be statistically significantly higher in cancer cases. No other statistically significant differences were found between the groups in terms of clinical factors. Since all consecutive cases that met the inclusion and exclusion criteria were included in the study, the ages of the cases in the cystadenoma and cystadenocarcinoma groups were found to be statistically significantly different.

**Figure 1:**
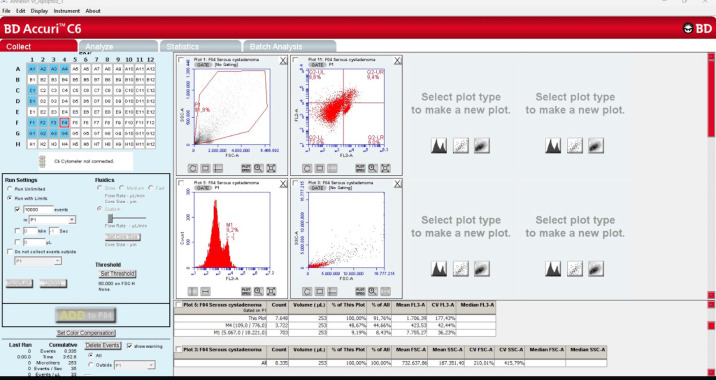
Sample flow cytometric analysis results for the aneuploidy cell ratio in a case of serous cystadenoma.

**Figure 2:**
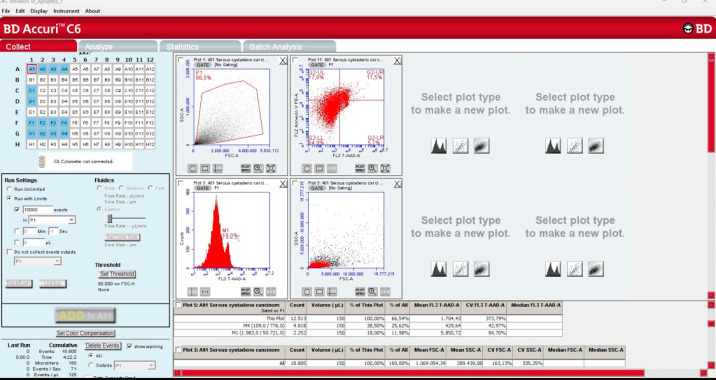
Sample flow cytometric analysis results for the aneuploidy cell ratio in a case of serous cystadenocarcinoma.

**Figure 3:**
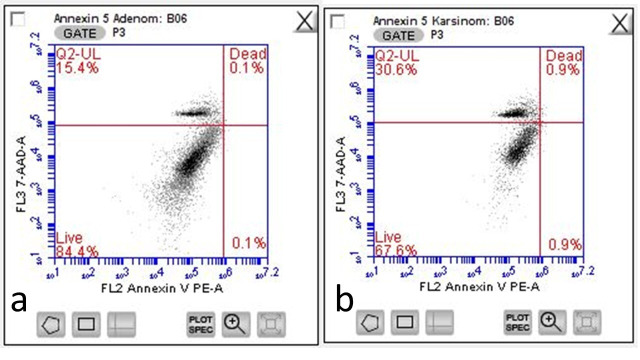
Sample flow cytometric analysis results for annexin V apoptotic index in cases of serous cystadenoma (**a**) and serous cystadenocarcinoma (**b**).

Table 2 shows a comparison of the G0/G1, S phase, G2/M stage, aneuploidy cell ratio (sample flow cytometric analysis results for aneuploidy cell ratio in cases of serous cystadenoma (Figure 1) and serous cystadenocarcinoma (Figure 2)), S phase, proliferative index, and annexin V apoptotic index-positive cell ratio (Figure 3, sample flow cytometric analysis result for annexin V apoptotic index in cases of serous cystadenoma (a) and serous cystadenocarcinoma (b)) in the serous cystadenoma and serous ovarian cystadenocarcinoma groups detected by flow cytometry. Since all the data in Table 2 met the parametric test assumptions, Student’s *t*-test was used for comparison. Accordingly, the S phase cell ratio, proliferative index, aneuploidy cell ratio, and annexin V apoptotic index ratio were found to be statistically significantly higher in the malignant group compared to the benign group. However, the G2/M phase cell ratio was found to be similar in both groups. The difference among the percentage of samples with aneuploidy in the benign (19/30, 16 of which were hyperdiploidy and 3 of which were hypodiploidy) and malignant groups (29/30, 24 of which were hyperdiploidy and 5 of which were hypodiploidy) was statistically significant (chi-square test was used for comparison, *p* = 0.0012).

### Student’s t-Test Was Used for Comparison

**Figure 4:**
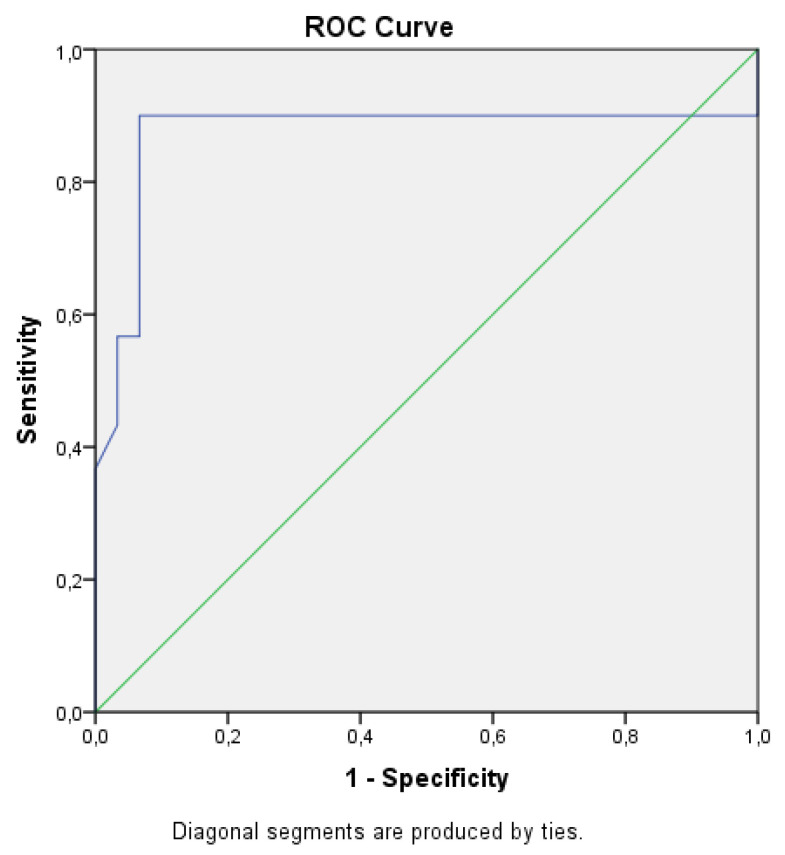
ROC analysis of the annexin V apoptotic index in terms of predicting serous ovarian carcinoma.

According to Pearson’s correlation analysis results, the annexin V apoptotic index showed a statistically significant positive correlation with the S phase cell ratio (r = 0.645, *p* < 0.001), aneuploidy cell ratio (r = 0.674, *p* < 0.001), and proliferative index (r = 0.462, *p* < 0.001). It showed a statistically significant negative correlation with the G0/G1 phase cell ratio (r = −0.619, *p* < 0.001). When the annexin V apoptotic index cutoff value was selected as 27.65%, the sensitivity was calculated as 90.0% and the specificity as 93.3% for predicting serous ovarian carcinoma (AUC 0.872, *p* <0.001, 95% CI 0.761–0.984, Figure 4). In addition, the flow cytometry analysis results did not show any variation based on the patient’s age.

## 4. Discussion

In this study, the flow cytometric analysis results (including the ratios of cells in the cell cycle stages) and apoptosis levels of serous ovarian cystadenoma cases were compared to serous cystadenocarcinoma cases. Cell division and the annexin V apoptotic index were found to be high in cancer cases. In addition, the cell ratio in the G0/G1 phase was found to be statistically significantly higher in benign cases. Although this supports the benign nature of the lesion, when compared to malignant cases, the cell ratios in the G2/M phase were found to be similar. This situation suggests that ovarian cystadenoma may be a precursor to ovarian cancer.

The cell cycle is a process characterized by cell division and the subsequent formation of two cells. It consists of four basic phases (G1, S, G2, M). In the G1 phase, the cell prepares for division; in the S phase, cell DNA is synthesized and copied; in the G2 phase, genetic material condensation and organization occur; and in the M phase, mitosis occurs and two daughter cells are formed. The flow cytometric analysis method, which determines the phase of cells by staining their DNA content, is used in the diagnosis of many medical diseases and cancers today [20]. There are few well-designed and analyzed studies in the literature that use flow cytometry for serous cystadenocarcinoma cases. Our study is valuable in terms of contributing to the literature because it includes homogeneous group data and comparative data with serous cystadenoma cases.

In a study that included a limited number of serous ovarian tumor cases, it was reported that flow cytometry results may have prognostic significance. Further, the study found that these tumors had increasing DNA proliferative activity as they progressed from benign to cancerous lesions. The proliferative index was shown to be 14.5 in cystadenoma and 23.2 in cystadenocarcinoma [21]. Similar findings were also found in our study.

It has been reported in many studies that cellular DNA content has prognostic importance in ovarian cancer and that its measurement would contribute to clinical practice [22,23]. However, the flow cytometry technique used in this measurement has not yet entered the routine diagnosis–follow-up scheme. In one study, aneuploidy was detected in 21 of 24 serous ovarian tumors and the proliferative index was found to be high in these cases [24]. Again, aneuploidy was shown to be present in 39–80% of serous tumors. It was also reported that the aneuploidy rate increased as the histological and nuclear grade increased [22]. In our study, the aneuploidy ratio, proliferation index, and S-phase fraction rate were found to be significantly higher in cancer cases.

According to the cell cycle phase research results of paraffin-embedded serous ovarian tumors (cystadenoma, borderline, cancer) and normal ovarian tissue using various DNA stains, all cell cycle phase activities increase dramatically during the progression of a normal ovary, to a cystadenoma, to a borderline tumor, and to cancer [25]. Although this finding has not been supported by literature data or extensive evidence-based medical data, it may support the claim that ovarian cystadenoma may be a precursor lesion for ovarian cancer. In our study, aneuploidy, the S phase, and the proliferative index ratio increased during the development of cystadenoma and the cancer process, while the G0/G1 ratio decreased. Similarly, G2/M phase activity, which provides information about cell genetic material condensation and mitosis phase activity, was found to be similar in both groups. This supports the argument that serous cystadenoma may be risky for cancer development in terms of cell division activity and that it may be a precursor lesion to serous cystadenocarcinoma.

It has been reported that social, genetic, and histopathological factors, as well as some new mutations (titin mutation, BAIAP3, IL34, WNT10A, NEU1, SLC44A4, HMOX1, TCN2, PES1, RP1-56J10.8, ABR, NCAM1, RP11-629G13.1, AC006372.4, and NPTXR in BOTS and hgOvCa, etc.) are associated with ovarian cancer survival and mortality [26,27]. In addition, it has been reported that tumor-associated macrophages found in the structures surrounding the tumor contribute to the development of ovarian cancer [28]. The presence of these cells in the apoptosis process shows that apoptosis contributes to the development of cancer. According to the results of current molecular studies, serous borderline ovarian tumor and low-grade ovarian serous cancer can transform into high-grade ovarian tumors by creating MDM2 amplification via p53 dysfunction [29]. Another recent study identified a new, distinct carcinogenesis pathway in serous ovarian tumors, namely the hippo signaling pathway genes [30]. All of these novel findings support the thesis that a distinct pathogenetic pathway operates in serous ovarian tumors. Further, they may support the existence of cancer development mediated by the serous ovarian cystadenoma–cancer pathway via different genetic mutations.

The exact mechanism of serous tumor carcinogenesis is unknown. Serous cystadenoma lesions are known to be benign, but a strong association with cancer has been reported in some cases [13]. This suggests that a new pathway may be involved in serous tumor carcinogenesis. Only one study to date has suggested that this could be called “metaneoplasia”. In that report, serous ovarian cancer cases occurring in a serous ovarian cyst against the background of a serous tubal intraepithelial carcinoma (STIC)-like lesion was reported. It was argued that the serous cystadenoma transformed into STIC-like lesions with genetic alterations, and that these lesions then transformed into ovarian high-grade cancer. This suggests the existence of a transformation process from cystadenoma to cancer with genetic mutations added in at each stage, just like the genetic hit theory in colon cancer. Therefore, cystadenoma may gradually transform into cystadenocarcinoma through genetic mutations. Whether this is the case or not can only be determined by research involving advanced genetic mutation analysis.

It is known that cell cycle markers are related to the behavior of the cell in the context of tumorigenesis. One of these, annexin V, is a marker of cell surface phosphatidylserine expression and is important for indicating apoptosis activity. Phosphatidylserine, which is located in the inner part of the plasma membrane in normal cells, becomes visible on the outer surface of the plasma membrane with the induction of apoptosis. It has been reported that annexin V expression in ovarian tumor cells in various stages of apoptosis is significantly increased compared to benign and normal cells [31]. It has also been reported in flow cytometry that annexin V is high in ascitic fluid in advanced-stage high-grade ovarian tumors and that it may be used as a biomarker of ovarian cancer [32]. Similarly, it was reported that apoptosis regulation was impaired in ovarian cancer [33] and annexin V-mediated apoptosis increased in serous ovarian cancer [34].

However, there are no sufficient studies in the literature that investigate the change in annexin V expression in serous cystadenocarcinoma cases compared to serous cystadenoma cases. In addition, according to literature data, the answer to the question of what the annexin V level will be in terms of a serous ovarian cancer diagnosis is not clear. In our study, it was concluded that the level of annexin V was significantly increased in serous cystadenocarcinoma cases compared to serous cystadenoma cases. Additionally, annexin V could be a biomarker with high sensitivity and specificity for the diagnosis of ovarian cancer when the annexin expression level is 27.65% and above.

Our study also provides new useful information that may be of clinical use. When the pathologist has difficulty diagnosing serous ovarian tumors or needs to support the diagnosis, they can use flow cytometry analysis, which is a fast, easy, and effective diagnostic method. Accordingly, annexin V staining can be requested and the diagnosis of ovarian cancer can be confirmed with high sensitivity and specificity. Our research can contribute effectively to the literature in this way.

According to the current review results, the amplification of over 30 defined growth stimulatory genes has been reported in 10% of epithelial ovarian tumors. It is known that these genes are significantly altered in advanced high-grade tumors compared to low-grade tumors [35]. Identifying the genes likely responsible for the transformation from serous cystadenoma to cystadenocarcinoma may offer cancer prevention and treatment options in the future by way of genetic technology and gene transfer. According to the results of the flow cytometry analysis of ovarian tumors, aneuploidy and the S-phase fraction ratio increased as the stage, grade, and Ca-125 level of the tumor increased [23]. This supports the argument that flow cytometry may be an important aid in the diagnosis and follow-up of serous tumors.

In oncology today, flow cytometric analysis is routinely used in determining intraoperative tumor margins in central nervous system tumors, head and neck tumors, breast cancer, and recently, in colon cancer surgery [36,37,38,39]. In addition, more specifically, flow cytometry is an important component when evaluating for suspected acute lymphoblastic leukemia lymphoblastic lymphoma, and it may be used to characterize the immunophenotype of lymphoblasts from peripheral blood, bone marrow, lymph nodes, or other tissue [40]. It is clear that flow cytometry is routinely indispensable in the diagnosis and treatment of leukemia/lymphoma according to the NCCN Clinical Practice Guidelines in Oncology 2025 [41]. However, there has been no sufficient literature data regarding its use in ovarian tumors. In this context, this retrospective study explored the effectiveness of routine flow cytometry use in ovarian tumors using paraffin-embedded ovarian tumor specimens. It was shown that flow cytometry use was effective in distinguishing between benign and malignant serous ovarian tumors. However, for the routine use of this method, comparative studies on fresh tissue and frozen section analysis are needed. In addition, the process of fixation could induce several artifacts in the tissue architecture. These were important limitations of our study. Flow cytometric analysis on fresh tissue provides results in less than 30 min. However, immunohistochemical analysis requires a few days and an experienced pathologist. Therefore, flow cytometric analysis on serous ovarian tumors has a high potential for routine use because it is fast, easy, and inexpensive.

This study offers new insights into the use of flow cytometry in gynecological cancer practice. However, it has limitations. The most important limitations of our study were the small number of cases and the lack of molecular genetic analysis to support our findings. Future studies with larger sample sizes are needed to confirm our findings.

## 5. Conclusions

Two important results emerged from this study. First, serous tumors of the ovary may progress from serous cystadenoma to serous cystadenocarcinoma by undergoing a “metaneoplasia” process that may involve complex genetic alterations. Second, the detection of annexin V expression in ovarian tumors by flow cytometric analysis is a cheap, fast, and easy diagnostic method that could be used in the diagnosis of ovarian cancer. Annexin V expression is an effective biomarker with high sensitivity and specificity that could be used in the diagnosis of serous ovarian cancer.

## Figures and Tables

**Table 1:** Comparison of the cases for select clinical data.

Clinical Data	Patients with Serous Cyst Adenoma (n = 30)	Patients with Serous Cystadenocarcinoma (n = 30)	*p*
Age ^a^	50.13 ± 15.01	57.80 ± 11.10	0.028
Gravida ^a^	3.96 ± 3.54	2.60 ± 2.06	0.181
Parity ^a^	3.04 ± 2.87	2.33 ± 1.95	0.404
Systemic disease (%) ^b^			0.399
Absent	63.3% (19)	76.7% (23)	
Present (hypertension, diabetes, thyroid disease, other)	36.7% (11)	23.3% (7)	
Surgery ^b^			0.001
TAH + USO or BSO/Staging	60.0% (18)	100.0% (30)	
Salpingo-oophorectomy	13.3% (4)	-	
Cystectomy	26.7% (8)	-	
FIGO stage			**-**
Stage I	-	33.3% (10)	
Stage II	-	26.7% (8)	
Stage III–IV	-	40.0% (12)	

TAH + BSO/USO; total abdominal hysterectomy + bilateral/unilateral salpingo-oophorectomy; ^a^ mean ± standard deviation; ^b^ percentage (number of cases in parenthesis) values are given. ^b^ Fisher’s exact test, chi-square test, or ^a^ Student’s *t*-test was used for comparison.

**Table 2:** Comparison of flow cytometric analysis results in the serous cystadenoma and serous ovarian cystadenocarcinoma groups.

	Patients with Serous Cyst Adenoma (n = 30)	Patients with Serous Cystadenocarcinoma (n = 30)	*p*
G0/G1 phase (%)	82.18 ± 2.25	73.09 ± 4.36	<0.001
G2/M phase (%)	8.55 ± 3.25	9.53 ± 1.96	0.163
S phase (%)	11.18 ± 1.66	18.25 ± 3.27	<0.001
Proliferative index (%)	18.89 ± 7.49	27.91 ± 4.36	<0.001
Aneuploidy cell ratio (%)	10.47 ± 1.96	18.24 ± 3.95	<0.001
Annexin V apoptotic index (%)	19.29 ± 5.40	34.04 ± 8.38	<0.001

## Data Availability

Any data set is available from the corresponding author upon request.

## Abbreviations

The following abbreviations are used in this manuscript:

STIC	Serous tubal intraepithelial carcinoma
TAH + BSO/USO	Total abdominal hysterectomy + bilateral/unilateral salpingo-oophorectomy
SPF	S-phase fraction
PI	Proliferative index
